# Triglyceride Glucose Index Associated With Arterial Stiffness in Chinese Community-Dwelling Elderly

**DOI:** 10.3389/fcvm.2021.737899

**Published:** 2021-09-13

**Authors:** Yongkang Su, Shuxia Wang, Jin Sun, Yan Zhang, Shouyuan Ma, Man Li, Anhang Zhang, Bokai Cheng, Shuang Cai, Qiligeer Bao, Ping Zhu

**Affiliations:** ^1^Medical School of Chinese People's Liberation Army, Beijing, China; ^2^Department of Geriatrics, The Second Medical Centre, National Clinical Research Center for Geriatric Diseases, Chinese People's Liberation Army General Hospital, Beijing, China; ^3^Department of Cadre Clinic, The First Medical Centre, Chinese People's Liberation Army General Hospital, Beijing, China; ^4^Department of Cardiology, The Second Medical Centre, National Clinical Research Center for Geriatric Diseases, Chinese People's Liberation Army General Hospital, Beijing, China

**Keywords:** triglyceride glucose index, insulin resistance, arterial stiffness, brachial-ankle pulse wave velocity, elderly

## Abstract

**Background:** The population of older adults is growing rapidly with the increasing pace of aging worldwide. The triglyceride glucose (TyG) index has been a convenient and reliable surrogate marker of insulin resistance (IR). This study aimed to determine the association between the TyG index and arterial stiffness assessed by brachial-ankle pulse wave velocity (baPWV) in Chinese older adults.

**Methods:** A total of 2,035 participants aged 60 years or above were enrolled. Demographic, anthropometric, and cardiovascular risk factors were collected. TyG index was calculated using ln (fasting triglycerides [mg/dL] × fasting glucose [mg/dL]/2). Arterial stiffness was measured using baPWV.

**Results:** The participants, with the mean [standard deviation (SD)] age of 71.32 (6.75) years, the female proportion of 39.65%, the mean (SD) baPWV of 1,998 (437) cm/s, and the mean (SD) TyG index of 8.86 (0.54), were divided into four groups according to TyG index quartiles. Age-adjusted baPWV presented an increasing trend according to TyG index quartiles. In the fully adjusted linear regression model, the baPWV increased 49 cm/s, with the 95% confidence interval (CI) from 24 to 75 cm/s, per-SD increase in the TyG index. In the fully-adjusted logistic regression model, the odds ratio (95% CI) of high baPWV (>75th percentile) was 1.32 (1.09, 1.60) for each SD increase in the TyG index. The generalized additive model analysis also confirmed the significant association of the TyG index with baPWV and high baPWV.

**Conclusion:** The TyG index is significantly associated with arterial stiffness assessed by baPWV in Chinese older adults.

## Introduction

The aging population is rapidly growing worldwide, and advanced aging has profound consequences on the health of older adults (1). Changes in vascular structure and function accompanying aging contribute to the genesis of arterial stiffness, one of the biomarkers of vascular aging (2). Arterial stiffness independently predicts the risk of cardiovascular events (3), the leading cause of global mortality (4).

Pulse wave velocity (PWV) generally is used to measure arterial stiffness (5). Carotid-femoral PWV, predominantly reflecting the stiffness of large-sized arteries, is typically used and validated in substantial studies (6). The baPWV is an indicator of stiffness of medium- to large-sized arteries. Consistent with the predictive value of carotid-femoral PWV, the baPWV is also independently associated with incidents of cardiovascular events, particularly in the Asia population (3, 7, 8). Additionally, measuring baPWV is relatively convenient, allowing its extensive use in clinical practices (7).

Insulin resistance (IR) typically refers to the condition that target tissues of insulin show decreased sensitivity to insulin-related metabolic actions. IR is characteristic of metabolic syndrome, obesity, and type 2 diabetes, and predicts the risk of cardiovascular disease (9, 10). TyG index, the product of fasting triglyceride and glucose, is a novel surrogate marker of IR and correlates well with the euglycemic-hyperinsulinemic clamp test considered the gold standard measure of IR (11–15). Moreover, the TyG index precedes the latter in evaluating IR, due to its simplicity, non-invasion, and inexpensiveness.

Several previous studies indicated that IR assessed by the TyG index is associated with arterial stiffness measured by the baPWV (16–22); however, discrepancies remain due to study population, sample size, and potential confounders. In this study, we investigated the association between the TyG index and the baPWV in a population of Chinese community-dwelling elderly.

## Materials and Methods

### Population

The population in the study derived from a community-based cross-sectional research conducted in the Wanshou Road Community of Haidian District in Beijing, China from September 2009 to June 2010. In brief, this survey used a two-stage stratified sampling method; a total of 2,162 persons aged from 60 to 95 years were selected as a representative sample of the elderly residing in the Wanshou Road Community. For our study, subjects with missing fasting triglyceride, fasting glucose, or baPWV were excluded (*n* = 63). Subjects treated with fibrates were also excluded due to its influence on the triglycerides (*n* = 7). We also excluded subjects with bilateral ankle-brachial index (ABI) < 0.9 (*n* = 57) that diminished the reliability of baPWV measurement (7). Ultimately, a total of 2,035 participants were included in the analyses. The study obtained the approval of the medical ethics committee of the Chinese PLA General Hospital.

### Demographic, Anthropometric, and Clinical Characteristics

Each participant was interviewed and completed a standardized questionnaire that included demographic factors, lifestyle, medical history, and medication information. Height, weight, and waist circumference were measured, and body mass index (BMI) was calculated as weight in kilograms divided by the square of height in meters. Blood pressure in the right arm was taken twice at 5-min intervals and the mean value was computed. Smoking was considered as three categorical variables for never, former, or current. Alcohol consumption was defined as one dichotomous variable for never/former or current. Hypertension was defined as diastolic blood pressure (DBP) of ≥90 mmHg, systolic blood pressure (SBP) of ≥140 mmHg, or current antihypertensive medication. Participants with a fasting plasma glucose (FPG) ≥ 7.0 mmol/l (126 mg/dL) or receiving hypoglycemic agents were diagnosed as diabetes mellitus (DM). Coronary heart disease (CHD) was identified by the self-reported information.

### Laboratory Assessment

Fasting blood specimens after overnight were collected for the test of serum lipids [triglycerides, total cholesterol (TC), high-density lipoprotein cholesterol (HDL-C), and low-density lipoprotein cholesterol (LDL-C)], glucose, uric acid (UA), and creatinine. The estimated glomerular filtration rate (eGFR) was calculated using the Chronic Kidney Disease Epidemiology Collaboration equation (23). The TyG index was calculated as ln (fasting triglycerides [mg/dL] × fasting glucose [mg/dL]/2) (11).

### Measurement of baPWV

Arterial stiffness was assessed by baPWV that was measured using an automated PWV/ABI analyzer (VP-1000; Colin Co., Ltd., Komaki, Japan), and ABI was measured simultaneously. In brief, brachial and post-tibial pressure waveforms were detected by cuffs wrapped around all four extremities connected to a plethysmographic sensor and an oscillometric sensor. Accordingly, the transit time between the brachial and post-tibial pressure waveforms (ΔTba) was determined, the traveled distance from the brachium to the ankle (La-Lb) was automatically calculated based on the participant's height, and baPWV was calculated using the formula (La-Lb)/ΔTba. Similarly, ABI was recorded as the ratio of the ankle systolic blood pressure divided by the brachial systolic blood pressure. Given the diminished reliability of baPWV measurement in subjects with severe atherosclerosis in lower extremities (7), we excluded subjects with bilateral ABIs < 0.9, for subjects with unilateral ABI < 0.9, we recorded contralateral baPWV, and for subjects with no ABI < 0.9, the mean of right and left baPWV was recorded for the analysis. Additionally, we defined high baPWV as a value greater than the 75th percentile of the baPWV values, which was 2,248 cm/s in this study (16, 19, 21, 22).

### Statistical Analysis

Distributions of characteristics of the participants were illustrated according to the TyG index quartiles. Continuous variables were described as mean ± SD, and frequency (%) was used for showing categorical variables. Differences of characteristics in different TyG quartiles were compared using analysis of variance or chi-square test. Age-adjusted the baPWV means and 95% confidence intervals (CI) of the TyG quartiles were calculated using analysis of covariance. The correlations between clinical parameters and baPWV, and the TyG index were determined using Pearson's correlation. The association between the TyG index and baPWV was examined using the multivariate linear regression model, and findings were reported by the beta coefficient (β) and 95% CI. The association between the TyG index and high baPWV was determined using the multivariable logistic regression model, and odds ratio (OR) and 95% CI were recorded as findings. As mentioned above, high baPWV was defined as a value > 75th percentile of baPWV (2,248 cm/s). Three models were built for the stepwise regression analyses. Model 1 was the unadjusted model, model 2 was the partially adjusted model that was controlled for age and sex, and model 3 was the fully adjusted model that was controlled for age, sex, BMI, waist circumference, SBP, DBP, TC, HDL-C, LDL-C, UA, eGFR, smoking status, drinking status, DM, hypertension, CHD, anti-platelet agents, anti-hypertensive agents, hypoglycemic therapy, and lipid-lowering therapy. We also standardized the TyG index, then put it into the regression models to determine the relationships between the increase of TyG per SD and baPWV, and high baPWV. In addition, the associations between the TyG index and baPWV, and high baPWV was evaluated and validated using the generalized additive model with the smoothness of fit. Moreover, subgroup and interaction analyses for the association between TyG index and high baPWV were performed to examine potential modifying factors. Given baPWV > 1,800 cm/s was a well-recognized criterion for high baPWV, we also verified the association between the TyG index and baPWV > 1,800 cm/s. Sensitivity analyses were also conducted to examine the association between the TyG index and baPWV in the non-diabetic subjects and subjects without taking hypoglycemic agents, respectively.

Two-sided *P* < 0.05 was considered statistically significant. All analyses were performed using the statistical software packages R (http://www.R-project.org, The R Foundation) and Empower Stats (http://www.empowerstats.com, X&Y Solutions, Inc., Boston, MA).

## Results

### Baseline Characteristics

Table 1 shows the characteristics of the participants, which were divided into four groups according to the TyG index quartiles. A total of 2,035 subjects were included in the analyses, with the mean (SD) cage of 71.32 (6.75) years, the female proportion of 39.65%, the mean (SD) baPWV of 1,998 (437) cm/s, and the mean (SD) TyG index of 8.86 (0.54). Subjects with higher TyG index were more likely to have elevated BMI, WC, SBP, DBP, FPG, TC, TG, and UA, and those with lower TyG index were more likely to have high HDL-C. In the high TyG index group, the proportion of women were relatively low, and the proportion of hypertension or diabetes mellitus was relatively high. In addition, participants with higher TyG index were prone to taking anti-hypertensive, hypoglycemic, and lipid-lowering medicines.

**Table 1:** Baseline characteristics of all participants according to TyG index quartile.

**Characteristics**	**Total**	**TyG index**				***P*-value**
		**Quartile1**	**Quartile2**	**Quartile3**	**Quartile4**	
*N*	2,035	509	508	509	509	
Age (years), mean ± SD	71.32 ± 6.75	71.70 ± 7.12	71.68 ± 7.01	71.13 ± 6.67	70.75 ± 6.15	0.069
Female, *n* (%)	806 (39.65%)	244 (47.94%)	214 (42.21%)	185 (36.35%)	163 (32.09%)	<0.001
BMI (kg/m^2^), mean ± SD	24.96 ± 3.39	23.55 ± 3.40	24.76 ± 3.27	25.40 ± 3.04	26.13 ± 3.29	<0.001
WC (cm), mean ± SD	88.05 ± 9.22	84.46 ± 9.08	87.25 ± 9.38	89.38 ± 8.45	91.11 ± 8.61	<0.001
Heart rate, bpm	71.87 ± 9.40	71.52 ± 9.92	71.36 ± 8.19	71.57 ± 8.85	73.02 ± 10.41	0.016
SBP (mmHg), mean ± SD	138.39 ± 19.43	135.07 ± 17.75	137.30 ± 19.15	137.64 ± 19.44	143.55 ± 20.33	<0.001
DBP (mmHg), mean ± SD	77.28 ± 9.76	75.51 ± 9.50	76.44 ± 9.27	77.04 ± 9.38	80.10 ± 10.26	<0.001
FPG (mmol/L), mean ± SD	6.04 ± 1.55	5.36 ± 0.68	5.71 ± 0.92	5.98 ± 1.04	7.10 ± 2.34	<0.001
Smoking status, *n* (%)						0.913
Never	1,488 (73.12%)	363 (71.32%)	372 (73.23%)	373 (73.28%)	380 (74.66%)	
Former	340 (16.71%)	87 (17.09%)	85 (16.73%)	86 (16.90%)	82 (16.11%)	
Current	207 (10.17%)	59 (11.59%)	51 (10.04%)	50 (9.82%)	47 (9.23%)	
Current drinking, *n* (%)	506 (24.86%)	143 (28.09%)	134 (26.38%)	108 (21.22%)	121 (23.77%)	0.060
TC (mmol/L), mean ± SD	5.26 ± 1.01	4.89 ± 0.94	5.22 ± 0.96	5.33 ± 0.95	5.61 ± 1.08	<0.001
HDL-C (mmol/L), mean ± SD	1.42 ± 0.39	1.64 ± 0.39	1.48 ± 0.35	1.34 ± 0.30	1.22 ± 0.36	<0.001
LDL-C (mmol/L), mean ± SD	3.21 ± 0.87	2.88 ± 0.77	3.24 ± 0.78	3.37 ± 0.79	3.36 ± 1.02	<0.001
TG (mmol/L), mean ± SD	1.68 ± 0.93	0.89 ± 0.18	1.29 ± 0.20	1.72 ± 0.30	2.83 ± 1.11	<0.001
UA (umol/L), mean ± SD	307.52 ± 87.42	297.52 ± 86.64	296.27 ± 85.04	312.38 ± 84.38	323.89 ± 90.76	<0.001
eGFR (mL/min/1.73 m^2^), mean ± SD	80.17 ± 14.54	80.86 ± 13.72	79.69 ± 15.05	80.02 ± 14.58	80.09 ± 14.79	0.628
TyG index	8.86 ± 0.54	8.22 ± 0.22	8.65 ± 0.09	8.99 ± 0.11	9.58 ± 0.34	<0.001
baPWV	1,998 ± 437	1,964 ± 450	1,976 ± 426	1,979 ± 406	2,074 ± 456	<0.001
CHD, *n* (%)	468 (23.00%)	101 (19.84%)	125 (24.61%)	129 (25.34%)	113 (22.20%)	0.147
Hypertension, *n* (%)	1,445 (71.29%)	311 (61.46%)	350 (69.31%)	366 (72.05%)	418 (82.28%)	<0.001
Diabetes mellitus, *n* (%)	477 (23.51%)	56 (11.00%)	83 (16.40%)	115 (22.68%)	223 (43.98%)	<0.001
Anti-platelet agents, *n* (%)	450 (22.16%)	93 (18.31%)	111 (21.89%)	122 (24.02%)	124 (24.41%)	0.075
Anti-hypertensive agents, *n* (%)	902 (44.63%)	182 (35.83%)	221 (43.68%)	244 (48.32%)	255 (50.80%)	<0.001
Hypoglycemic therapy, *n* (%)	333 (16.41%)	44 (8.64%)	67 (13.24%)	79 (15.58%)	143 (28.21%)	<0.001
Lipid-lowering therapy, *n* (%)	212 (10.65%)	37 (7.49%)	49 (9.88%)	56 (11.18%)	70 (14.00%)	0.009
High baPWV	508 (24.96%)	111 (21.81%)	117 (23.03%)	121 (23.77%)	159 (31.24%)	0.002
baPWV > 1,800 cm/s	1,282 (63.00%)	302 (59.33%)	311 (61.22%)	312 (61.30%)	357 (70.14%)	0.002

*TyG, triglyceride glucose; SD, standard deviation; BMI, body mass index; WC, waist circumference; SBP, systolic blood pressure; DBP, diastolic blood pressure; FPG, fasting plasm glucose; TC, total cholesterol; HDL-C, high-density lipoprotein cholesterol; LDL-C, low-density lipoprotein cholesterol; TG, triglyceride; UA, uric acid; eGFR, estimated glomerular filtration rate; CHD, coronary heart disease; baPWV, brachial-ankle pulse wave velocity*.

**Figure 1:**
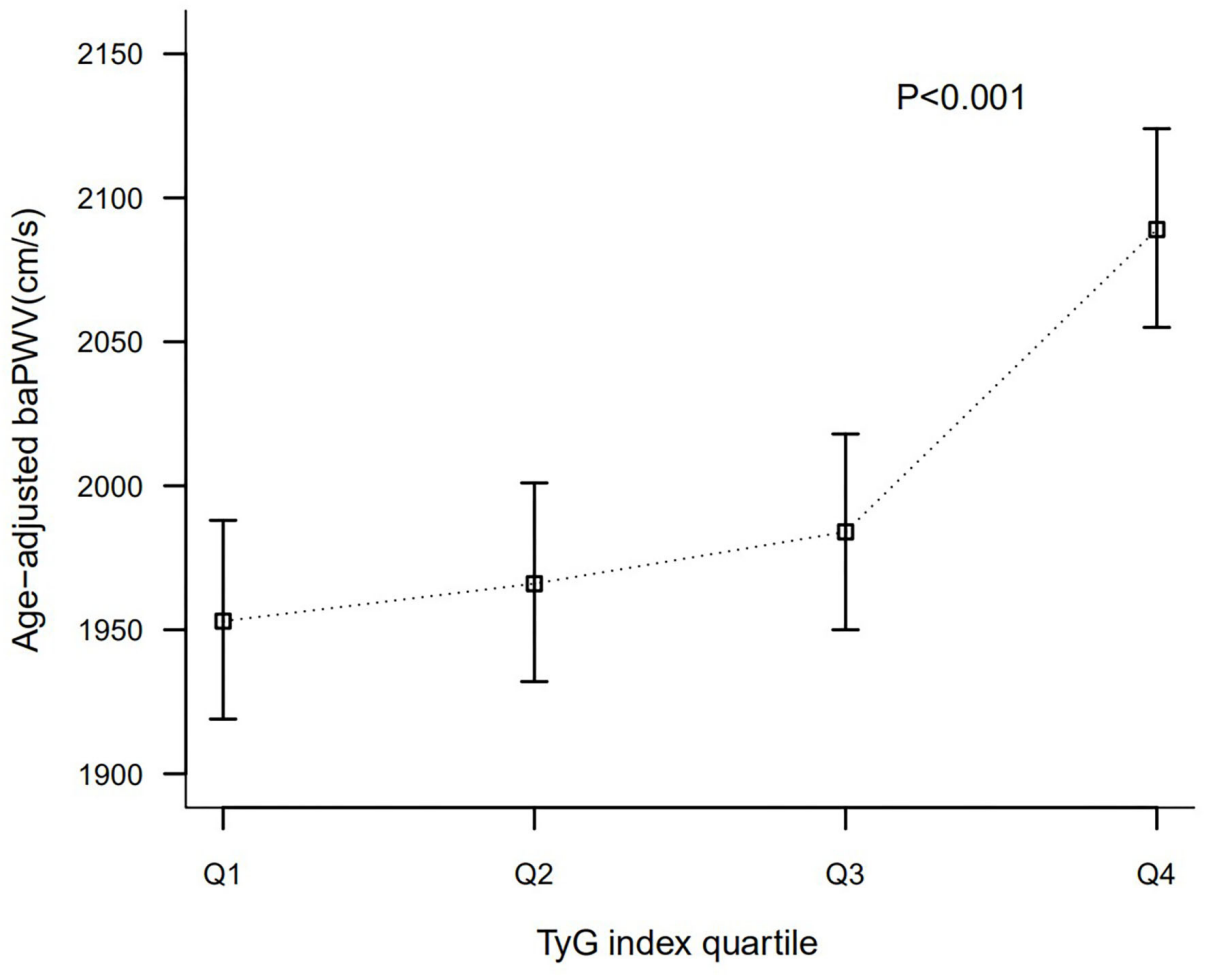
Comparison of baPWV according to TyG index quartile after adjusted for age.

As shown in Figure 1, age-adjusted baPWV presented an increasing trend according to TyG index quartiles. Means (95% CIs) of age-adjusted baPWV of different TyG groups (Quartile1–4) were 1,953 (1,919, 1,988), 1,966 (1,932, 2,001), 1,984 (1,950, 2,018), and 2,089 (2,055, 2,124) cm/s, respectively (*P* < 0.001).

### Correlation Between Clinical Variables and baPWV, TyG Index

After adjusted for age and sex, baPWV was significantly correlated with TyG index, BMI, SBP, DBP, TC, and LDL-C, and TyG index was significantly correlated with BMI, waist circumference, SBP, DBP, TC, HDL-C, LDL-C, urine acid, and eGFR (Table 2).

**Table 2:** Age- and sex-adjusted correlation between TyG index, baPWV, and clinical variables.

**Variables**	**baPWV**		**TyG index**	
	***r***	***P*-value**	***r***	***P*-value**
BMI (kg/m^2^)	−0.056	0.011	0.285	<0.001
WC (cm)	0.002	0.938	0.327	<0.001
SBP (mmHg)	0.405	<0.001	0.167	<0.001
DBP (mmHg)	0.374	<0.001	0.197	<0.001
TC (mmol/L)	0.053	0.018	0.263	<0.001
HDL-C (mmol/L)	−0.029	0.198	−0.428	<0.001
LDL-C (mmol/L)	0.047	0.036	0.160	<0.001
UA (umol/L)	−0.001	0.970	0.171	<0.001
eGFR (mL/min/1.73 m^2^)	0.016	0.469	−0.051	0.022
TyG index	0.132	<0.001	–	–

*TyG, triglyceride glucose; baPWV, brachial-ankle pulse wave velocity; BMI, body mass index; WC, waist circumference; SBP, systolic blood pressure; DBP, diastolic blood pressure; FPG, fasting plasm glucose; TC, total cholesterol; HDL-C, high-density lipoprotein cholesterol; LDL-C, low-density lipoprotein cholesterol; UA, uric acid; eGFR, estimated glomerular filtration rate*.

### Association Between TyG Index and baPWV

**Figure 2:**
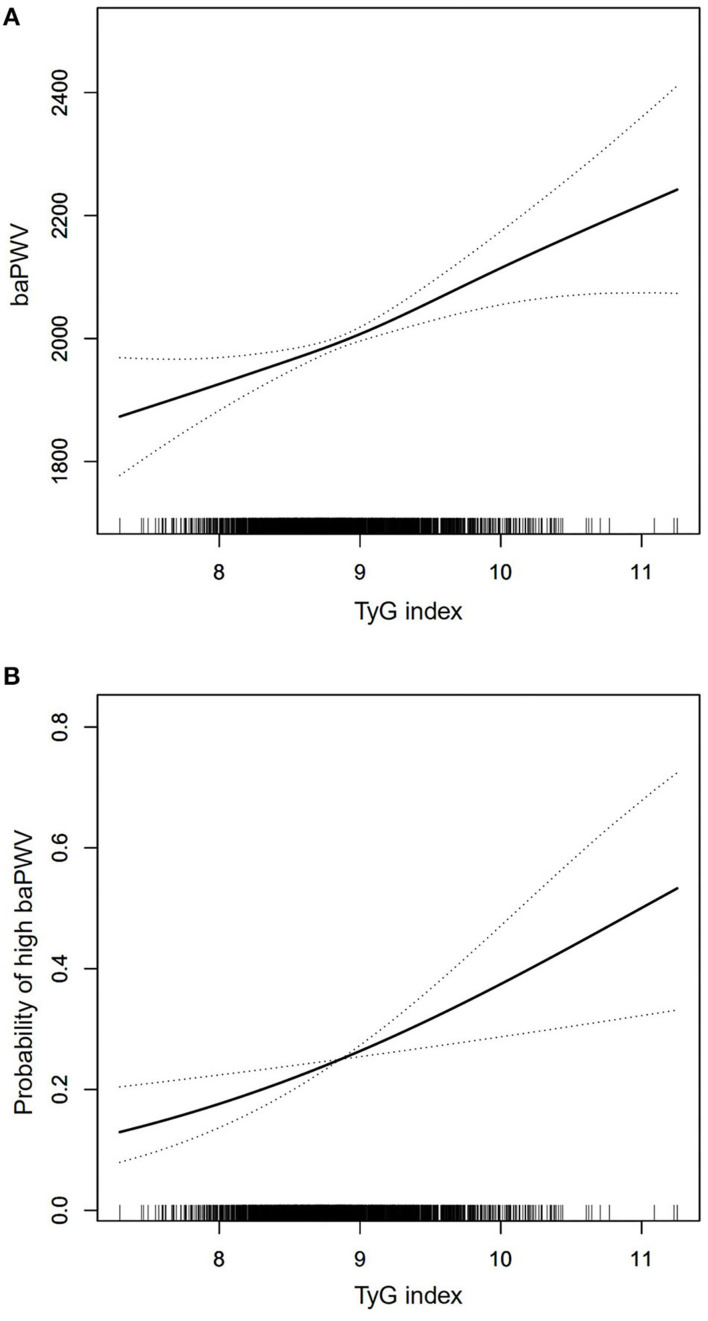
Generalized additive model plot for the relationship of TyG index with baPWV **(A)** and high baPWV **(B)** after adjusted for age, sex, BMI, waist circumference, SBP, DBP, TC, HDL-C, LDL-C, eGFR, smoking status, drinking status, CHD, hypertension, diabetes mellitus, anti-platelet agents, anti-hypertensive agents, hypoglycemic therapy, and lipid-lowering therapy.

Table 3 exhibited that baPWV had a change of 49 (95% CI: 24, 75) cm/s per SD increase in TyG index according to the fully adjusted linear regression model. Based on the quartiles, TyG index as a categorized variable entered into the models, and the first quartile was considered as the reference; in the fully adjusted model, quartile4 of TyG index was significantly associated with an increase of 86 (95% CI: 24, 147) cm/s in baPWV compared to quartile 1 of TyG index, and we found an increasing trend of the β coefficients of baPWV across TyG index quartiles (*P* for trend = 0.012). The generalized additive model analysis also determined the significant positive association between the TyG index and baPWV (Figure 2A).

**Table 3:** Linear regression analyses for the association between TyG index and baPWV.

**TyG index**	**β** **(95% CI), baPWV (cm/s)**		
	**Model 1^a^**	**Model 2^b^**	**Model 3^c^**
Per SD increase	50 (31, 69)^***^	53 (36, 70)^***^	49 (24, 75)^***^
Quartile1	0 (Reference)	0 (Reference)	0 (Reference)
Quartile2	13 (−41, 66)	9 (−39, 58)	13 (−33, 59)
Quartile3	16 (−38, 69)	23 (−26, 72)	24 (−25, 74)
Quartile4	111 (58, 164)^***^	126 (77, 175)^***^	86 (24, 147)^**^
P for trend	*p* < 0.001	*p* < 0.001	0.012

*TyG, triglyceride glucose; baPWV, brachial-ankle pulse wave velocity; SD, standard deviation; CI, confidence interval*.

a *Adjusted for none*.

b *Adjusted for age and sex*.

c *Adjusted for age, sex, BMI, waist circumference, SBP, DBP, TC, HDL-C, LDL-C, UA, eGFR, smoking status, drinking status, CHD, hypertension, diabetes mellitus, anti-platelet agents, anti-hypertensive agents, hypoglycemic therapy, and lipid-lowering therapy*.

* *P < 0.05*.

** *P < 0.01*.

*** *P < 0.001*.

### Association Between TyG Index and High baPWV

As shown in Table 4, for each SD increase in the TyG index, the OR (95% CI) of high baPWV was 1.32 (1.09, 1.60) in the fully adjusted regression model. The fully adjusted ORs (95% CIs) for high baPWV for subjects in quartile2, quartile3, and quartile4 were 1.18 (0.82, 1.68), 1.27 (0.87, 1.87), and 1.78 (1.12, 2.81), respectively. The increasing risk of high baPWV from quartile1 to quartile4 was statistically significant (*P* for trend = 0.019). Similarly, the significant association between the TyG index and high baPWV was also found in generalized additive model analysis (Figure 2B).

**Table 4:** Logistic regression analyses for the association between TyG index and high baPWV.

**TyG index**	**OR (95% CI)**		
	**Model 1^a^**	**Model 2^b^**	**Model 3^c^**
Per SD increase	1.24 (1.12, 1.36)^***^	1.28 (1.15, 1.42)^***^	1.32 (1.09, 1.60)^**^
Quartile1	1 (Reference)	1 (Reference)	1 (Reference)
Quartile2	1.07 (0.80, 1.44)	1.07 (0.78, 1.46)	1.18 (0.82, 1.68)
Quartile3	1.12 (0.83, 1.50)	1.17 (0.86, 1.59)	1.27 (0.87, 1.87)
Quartile4	1.63 (1.23, 2.16)^***^	1.83 (1.35, 2.47)^***^	1.78 (1.12, 2.81)^*^
P for trend	*p* < 0.001	*p* < 0.001	0.019

*TyG, triglyceride glucose; baPWV, brachial-ankle pulse wave velocity; SD, standard deviation; OR, odds ration; CI, confidence interval*.

a *Adjusted for none*.

b *Adjusted for age and sex*.

c *Adjusted for age, sex, BMI, waist circumference, SBP, DBP, TC, HDL-C, LDL-C, UA, eGFR, smoking status, drinking status, CHD, hypertension, diabetes mellitus, anti-platelet agents, anti-hypertensive agents, hypoglycemic therapy, and lipid-lowering therapy*.

* *P < 0.05*.

** *P < 0.01*.

*** *P < 0.001*.

### Subgroup Analyses for Association Between TyG Index and High baPWV

**Figure 3:**
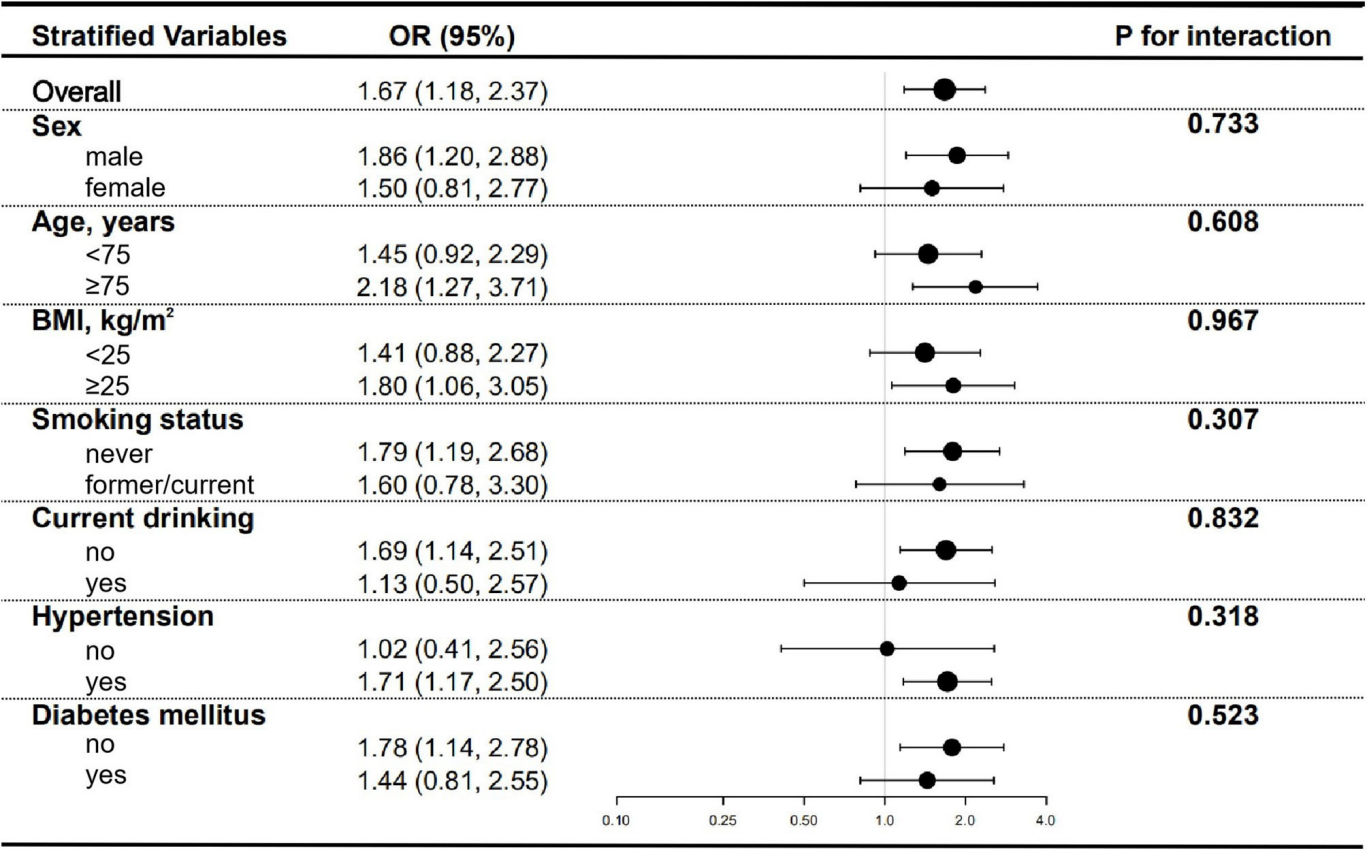
Subgroup analyses for the association between TyG index and high baPWV. Adjusted for age, sex, BMI, waist circumference, SBP, DBP, TC, HDL-C, LDL-C, eGFR, smoking status, drinking status, CHD, hypertension, diabetes mellitus, anti-platelet agents, anti-hypertensive agents, hypoglycemic therapy, and lipid-lowering therapy except for the stratified variable.

Several stratified analyses were performed to further examine the relationship between the TyG index and high baPWV. As shown in Figure 3, the significantly association between TyG index and high baPWV in the subgroups of men [OR (95% CI), 1.86 (1.20, 2.88)], those aged ≥ 75 years [OR (95% CI), 2.18 (1.27, 3.71)], those with BMI ≥ 25 kg/m^2^ [OR (95% CI), 1.80 (1.06, 3.05)], those never-smoking [OR (95% CI), 1.79 (1.19, 2.68)], those not currently drinking [OR (95% CI), 1.69 (1.14, 2.51)], those with hypertension [OR (95% CI), 1.71 (1.17, 2.50)], and those without diabetes mellitus [OR (95% CI), 1.78 (1.14, 2.78)], were consistent with the overall [OR (95% CI), 1.67 (1.18, 2.37)]. Nevertheless, the relationship between TyG index and high baPWV was not statistically significant in subgroups of females, those <75 years of age, those with BMI < 25 kg/m^2^, those formerly or currently smoking, those currently drinking, those without hypertension, and those with diabetes mellitus, which was probably attributed to the small sample size. And there were no interactions that were found in all subgroup analyses.

The association between the TyG index and baPWV > 1,800 cm/s was also determined. As shown in Supplementary Table 1, the OR (95% CI) of baPWV > 1,800 cm/s was 1.41 (1.17, 1.70) per SD increment in the TyG index, and the increasing trend of risk of baPWV > 1,800 cm/s from quartile1 to quartile4 was significant (*P* for trend = 0.004). No interactions were found in the stratified analyses for the association between the TyG index and baPWV > 1,800 cm/s (Supplementary Figure 1). In addition, similar results were found when the sensitivity analyses were conducted in the non-diabetic subjects and subjects without taking hypoglycemic agents, respectively (Supplementary Tables 2, 3).

## Discussion

In the present study, we confirmed that the TyG index was significantly associated with baPWV and arterial stiffness evaluated by the specific cut-off of baPWV in the Chines elderly. Our findings further supported the increasing evidence that the predictive value of TyG index to arterial stiffness, and validated this association available in the elderly population.

Our study showed that the mean baPWV was 1,998 cm/s in the community-dwelling elderly of mean age of 71.32 years. In the Northern Shanghai Study, Zhao et al. reported that the older adults of mean age of 71.5 years had the mean baPWV of 1,871.9 cm/s (18). Yiming et al. explored the reference values of baPWV in Han subjects aged 15–88 years in Xinjiang, China, and reported that the mean baPWV of aged above 60 years was 1,630 cm/s in the participants without smoking, hypertension, dyslipidemia, and diabetes, and ranged from 1,780 to 1,960 cm/s in the subjects with hypertension (24). It was well-recognized that age and blood pressure were predominant determinants of arterial stiffness (7). By contrast, the prevalence of hypertension was 71.29% in our study, which was higher than 65.84 and 39.1% in the other two studies. The difference in the prevalence of hypertension in the three studies was consistent with the distribution of prevalence of hypertension in China, which was that the northern was higher than the southern, the prevalence was higher in large and medium-sized cities, and the residents in Beijing had the highest prevalence (25). Accordingly, we speculated that discrepancies in the distribution of baPWV in the three studies were likely attributed to the different prevalence of hypertension. Given the limited samples of the three studies, further national sample studies were necessary to elaborate the distribution of baPWV among Chinese populations.

As previously mentioned, the TyG index is the transformation of fasting triglyceride and glucose and an admissible and reliable surrogate indicator of insulin resistance; baPWV, reflecting the stiffness of medium- to large-sized arteries, was a simple and reproducible marker of arterial stiffness. Previous studies showed that arterial stiffness was associated with conventional risk factors for cardiovascular disease, including advanced age and high blood pressure (7). Our study found that traditional cardiometabolic risk factors were significantly associated with the TyG index, which corresponded to previous studies and may partly explain the association between the TyG index and arterial stiffness.

Several antecedents have investigated the relationship between the TyG index and baPWV. Lee et al. and Won et al. showed the independent association between TyG index and arterial stiffness measured by baPWV in Korean adults (16, 17). Guo et al. also reported that the TyG index was independently associated with high baPWV after controlled for classical cardiovascular risk factors in a Chinese population (21). Unfortunately, the subjects in all three studies above were predominantly middle-aged, not limited to older adults. Furthermore, no stratified analyses were conducted to further validated and explored the association between the TyG index and baPWV. It is well-known that the pace of the population aging worldwide is accelerating, resulting in the dramatic increase of the elderly population that has become the primary target of health care (26). Given the potential prognostic significance of the TyG index and baPWV, a clear understanding of the association between the two in the elderly is essential.

To our best knowledge, few studies to date have examined the relationship between the TyG index and arterial stiffness in the elderly. In the Atherosclerosis Risk in Communities Study, Poon et al. reported that for each SD increase in the TyG index, carotid-femoral pulse wave velocity (cfPWV) had a change of 32 (95% CI: 22, 42) cm/s, and the OR (95% CI) of high cfPWV (>75th percentile) was 1.21 (1.11, 1.32), with that arterial stiffness was assessed using cfPWV > 75th percentile (27). Zhao et al. revealed that the high TyG index was significantly positively associated with risk of arterial stiffness in one elderly population ocean (SD) age 71.5 (6.2) years (18), with that the TyG index served as the categorized variable based on the quartiles, and arterial stiffness was defined as baPWV > 1,800 cm/s. It is well-recognized that cfPWV and baPWV, as the arterial stiffness index, have a positive correlation (28, 29). However, by contrast with similar studies, some improvements probably enhance reliability and validity, including exclusion of participants with ABI < 0.9, continuous and categorical variable types of the TyG index, trend test analyses, generalized additive models, subgroup analyses, interaction analyses, and sensitivity analyses. In the present study conducted in community-based older adults excluded from peripheral arterial disease, we also found the independent and positive relationship between the TyG index and the increased baPWV regardless of the variable pattern of the TyG index. The findings of the stratified analyses seem to be consistent if we paid no more attention to the significance of statistics that was prone to be affected by the sample size. Unfortunately, we found no potential modifiers on the association between the TyG index and the baPWV.

Although our study further confirms the association between the TyG index and high baPWV, the underlying mechanism linking TyG with arterial stiffness is incompletely understood. Substantial evidence has demonstrated the relationship between the TyG index and insulin resistance, and the TyG index has been suggested as a reliable surrogate marker of IR. Accordingly, we speculate that IR may play a key role in connecting the TyG index with arterial stiffness. Subclinical vascular disease is associated with insulin resistance, and vascular functional and structural damage resulted from IR includes the reduced distensibility of the arterial wall that is arterial stiffness (30–32). Hyperinsulinemia resulting from insulin resistance contributes to the emergence of endothelial cell dysfunction, followed by the genesis of the decrease in bioavailable nitric oxide, which leads to endothelial-dependent vascular dysfunction, including impaired vascular relaxation, inflammation, vascular remodeling, and overt fibrosis (10, 33–35). Ultimately, all of these alterations in vascular function and structure collectively stiffen the arterial wall.

Several limitations are in our study. First, we cannot establish the causal relationship between the TyG index and the baPWV due to the cross-sectional study design. Second, the level of fasting insulin was not detected, resulting in that we could not calculate the homeostatic model assessment of insulin resistance (HOMA-IR), which is typically used to assess IR in clinical practices. However, previous studies have proved that the TyG index is strongly associated with HOMA-IR, and the TyG index may have a better predictive value for IR than HOMA-IR (36, 37). Third, the subjects in this study were recruited from one Chinese urban community, which probably limited the generalizability of the findings. Lastly, we could not adjust some potential confounders that could affect the results, including dietary habits and physical activity, although a broad spectrum of adjusted factors entered into the analyses. Even so, our study also has significance in itself. Our findings give support to the significant association between the TyG index and baPWV and expand the population of applicability.

## Conclusion

Our study demonstrated that the TyG index was significantly associated with arterial stiffness measured by baPWV in Chinese older adults. Our findings suggest that the TyG index, a simple and effective indicator, may have some significance in health care for the elderly, and further studies are necessary to explore and validate the practical value of the TyG index.

## Data Availability Statement

The datasets used in the study are available from the corresponding author upon reasonable request.

## Ethics Statement

The studies involving human participants were reviewed and approved by the medical Ethics Committee of the Chinese PLA General Hospital. The patients/participants provided their written informed consent to participate in this study.

## Author Contributions

YS and SW designed the study, conducted the data analyses, drafted the manuscript, and revised the manuscript. JS, AZ, BC, SC, and QB assisted with the survey. YZ, SM, and ML reviewed the manuscript. PZ designed the study and revised the manuscript. All authors read and approved the final manuscript.

## Funding

This study was supported by the National Key R&D Program of China (2020YFC2008900) and the Logistics Scientific Research Project of the Chinese PLA (19BJZ30).

## Conflict of Interest

The authors declare that the research was conducted in the absence of any commercial or financial relationships that could be construed as a potential conflict of interest.

## Publisher's Note

All claims expressed in this article are solely those of the authors and do not necessarily represent those of their affiliated organizations, or those of the publisher, the editors and the reviewers. Any product that may be evaluated in this article, or claim that may be made by its manufacturer, is not guaranteed or endorsed by the publisher.
