# Supplementary material for: Triglyceride Glucose Index Associated With Arterial Stiffness in Chinese Community-Dwelling Elderly
Source: Front Cardiovasc Med. 2021 Sep 13;8:737899. doi: 10.3389/fcvm.2021.737899 (PMC8473610; doi:10.3389/fcvm.2021.737899)
Supplement: Supplementary file 1 [file Data_Sheet_1.docx]

Supplementary Material

##

**Table 1.** Logistic regression analyses for the association between TyG index and baPWV>1800cm/s

| **TyG index** | **OR (95%CI)** | | |
| --- | --- | --- | --- |
|  | **Model 1^a^** | **Model2^b^** | **Model 3^c^** |
| Per SD increase | 1.22 (1.11, 1.34) *** | 1.28 (1.16, 1.42) *** | 1.41 (1.17, 1.70) ** |
| Quartile1 | 1(Reference) | 1(Reference) | 1(Reference) |
| Quartile2 | 1.08 (0.84, 1.39) | 1.09 (0.83, 1.44) | 1.19 (0.86, 1.65) |
| Quartile3 | 1.09 (0.84, 1.40) | 1.16 (0.88, 1.52) | 1.34 (0.94, 1.91) |
| Quartile4 | 1.61 (1.24, 2.09) *** | 1.87 (1.41, 2.49) *** | 2.00 (1.28, 3.14) ** |
| P for trend | p<0.001 | p<0.001 | 0.004 |

Abbreviation: TyG, triglyceride glucose; baPWV, brachial-ankle pulse wave velocity; SD, standard deviation; OR, odds ration; CI, confidence interval.

^a^ adjusted for none.

^b^ adjusted for age and sex.

^c^ adjusted for age, sex, BMI, waist circumference, SBP, DBP, TC, HDL-C, LDL-C, UA, eGFR, smoking status, drinking status, CHD, hypertension, diabetes mellitus, anti-platelet agents, anti-hypertensive agents, hypoglycemic therapy, and lipid-lowering therapy.

*P<0.05

**P<0.01

***P<0.001


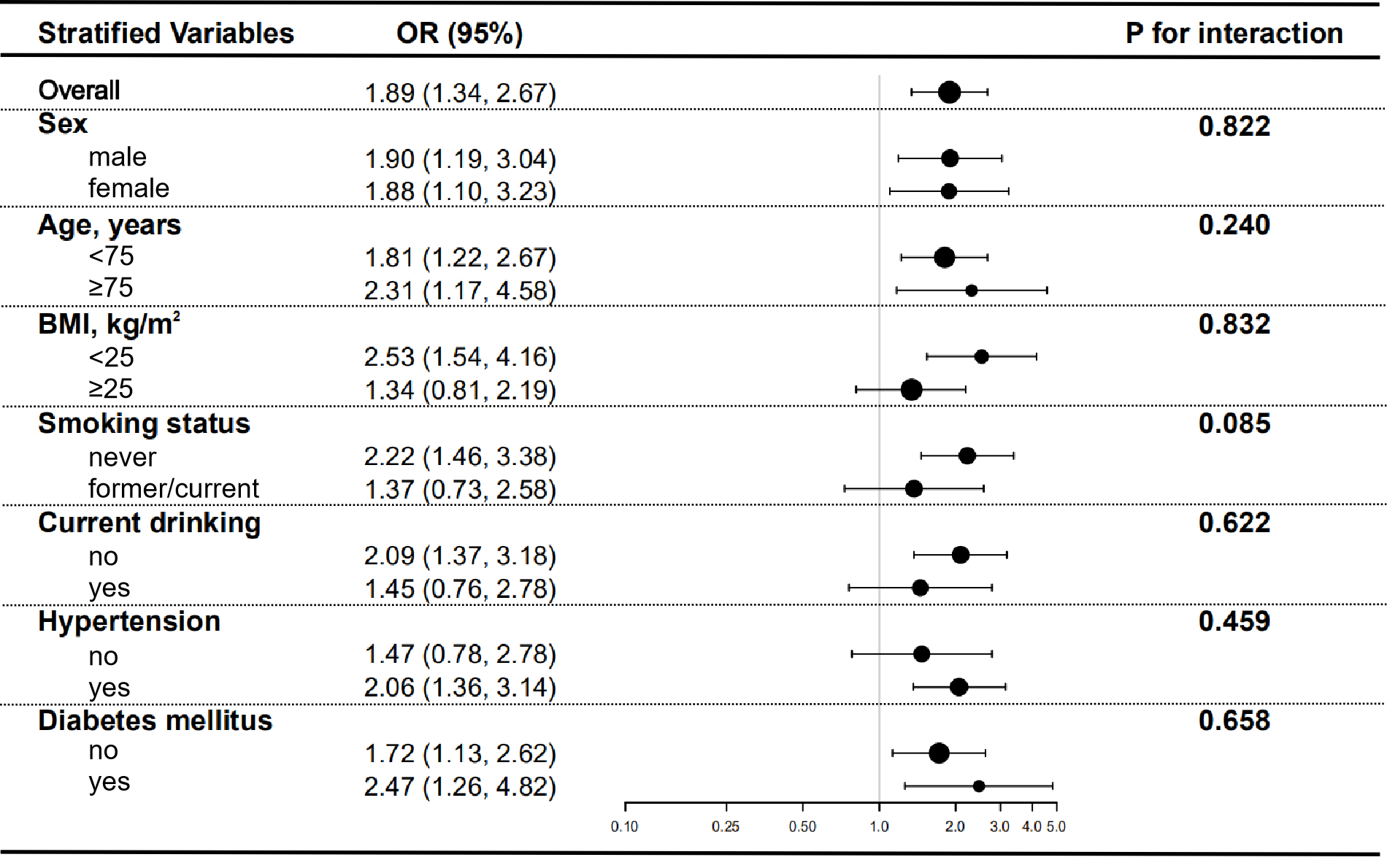


**Figure 1.** Subgroup analyses for the association between TyG index and baPWV >1800cm/s. Adjusted for age, sex, BMI, waist circumference, SBP, DBP, TC, HDL-C, LDL-C, eGFR, smoking status, drinking status, CHD, hypertension, diabetes mellitus, anti-platelet agents, anti-hypertensive agents, hypoglycemic therapy, and lipid-lowering therapy except for the stratified variable

**Table 2.** Association between TyG index and high baPWV / baPWV >1800cm/s in the non-diabetic subjects

| **TyG index** | **OR (95%CI)** | |
| --- | --- | --- |
|  | **Model 3 for high baPWV** | **Model 3 for baPWV >1800cm/s** |
| Per 1 unit increase | 1.70 (1.16, 2.47) ** | 1.79 (1.24, 2.61) ** |
| Quartile1 | 1(Reference) | 1(Reference) |
| Quartile2 | 1.36 (0.89, 2.08) | 1.19 (0.82, 1.74) |
| Quartile3 | 1.42 (0.96, 2.10) | 1.32 (0.92, 1.89) |
| Quartile4 | 1.92 (1.17, 3.15) ** | 1.80 (1.13, 2.86) * |
| P for trend | 0.016 | 0.027 |

Abbreviation: TyG, triglyceride glucose; baPWV, brachial-ankle pulse wave velocity; SD, standard deviation; OR, odds ration; CI, confidence interval.

Adjusted for age, sex, BMI, waist circumference, SBP, DBP, TC, HDL-C, LDL-C, UA, eGFR, smoking status, drinking status, CHD, hypertension, diabetes mellitus, anti-platelet agents, anti-hypertensive agents, hypoglycemic therapy, and lipid-lowering therapy.

*P<0.05

**P<0.01

***P<0.001

**Table 3.** Association between TyG index and high baPWV / baPWV >1800cm/s in the subjects without taking hypoglycemic agents

| **TyG index** | **OR (95%CI)** | |
| --- | --- | --- |
|  | **Model 3 for high baPWV** | **Model 3 for baPWV >1800cm/s** |
| Per 1 unit increase | 1.78 (1.14, 2.78) * | 1.72 (1.13, 2.62) * |
| Quartile1 | 1(Reference) | 1(Reference) |
| Quartile2 | 1.26 (0.81, 1.97) | 1.20 (0.81, 1.79) |
| Quartile3 | 1.33 (0.88, 2.01) | 1.32 (0.90, 1.93) |
| Quartile4 | 2.01 (1.19, 3.40) ** | 1.81 (1.11, 2.97) * |
| P for trend | 0.015 | 0.037 |

Abbreviation: TyG, triglyceride glucose; baPWV, brachial-ankle pulse wave velocity; SD, standard deviation; OR, odds ration; CI, confidence interval.

Adjusted for age, sex, BMI, waist circumference, SBP, DBP, TC, HDL-C, LDL-C, UA, eGFR, smoking status, drinking status, CHD, hypertension, diabetes mellitus, anti-platelet agents, anti-hypertensive agents, hypoglycemic therapy, and lipid-lowering therapy.

*P<0.05

**P<0.01

***P<0.001
